# Reduced Invasiveness of Cardiopulmonary Bypass: The Mini-Circuit and the Micro-Cardioplegia

**DOI:** 10.3390/jcdd10070290

**Published:** 2023-07-07

**Authors:** Thierry Carrel

**Affiliations:** 1 Departement of Cardiac Surgery, University of Zürich, CH-8006 Zürich, Switzerland; thierry.carrel@gmail.com; 2 Department of Cardiac Surgery, University Hospital Basel, CH-4052 Basel, Switzerland

**Keywords:** cardiopulmonary bypass, minimal invasive, cardioplegia, systemic inflammation

## Abstract

The aim of cardiopulmonary bypass is the maintenance of a sufficient whole body perfusion and gas exchange during open or closed heart surgery procedure (coronary artery bypass grafting, valve repair and replacement, surgical intervention on the ascending aorta and/or aortic arch, repair of congenital malformations, and finally implantation of ventricular assist devices or cardiac transplantation). The main components of cardiopulmonary bypass are the pump that supplies the circulation and the oxygenator that regulates gas exchange. However, even though this technology has been extensively developed and improved over the last decades, one of the major drawbacks—which is the fact that blood has to flow through tubing systems with foreign surfaces—persists so far. Nevertheless, interesting innovations have been made more recently in order to better control the side-effects that culminate into a major activation of the coagulation and inflammatory systems: among them, miniaturization of the circuits, together with reduction of the priming volume and a simplified cardioplegia concept. All of these lead to a significant decrease of hemodilution and thereby a significant reduction of volume overload during surgery. In this brief review we will present some of these most interesting topics around minimized circuits and the simplified low-volume cardioplegia and discuss their potential benefits on the clinical outcome.

## 1. Introduction

Cardiopulmonary bypass (CPB) support is required when the heart has to be empty for an intracardiac repair, when a mechanical arrest is required or when manipulations on the heart (luxation) need support for the systemic circulation. Another condition is the case where moderate or deep hypothermia is necessary to allow circulatory arrest, for instance when the aortic arch has to be repaired and complex congenital surgeries performed. Although it has been introduced in the late fifties and further developed in the sixties and seventies to repair congenital heart diseases, to bypass coronary arteries, to repair or replace heart valves, to treat aortic pathologies and last but not least to transplant hearts, CPB technology did not receive too much attention to reduce unintended adverse effects, often referred to as the postperfusion syndrome, or the whole body inflammatory response. These adverse effects may result in different degrees of organ dysfunction and therefore in the need for prolonged intensive care treatment and longer hospital stay.

To allow work on or into the heart, deoxygenated blood is drained into an extracorporeal reservoir, usually made of compressible polyvinyl bags or hard-shell plastic, which results in blood-air interface. Once it enters into the extracorporeal bypass circuit, the blood encounters a number of abnormal elements, like air bubbles, fibrin, tissue debris, platelets thrombi and defoaming agents. From the reservoir, the blood is directed to the oxygenator and runs thereafter into a heat exchanger that regulates the blood temperature into the circuit (and therefore also in the body). Finally, the blood is filtered through nylon or polyester screens and pumped back into the patient at a controlled flow rate either by a roller pump or a centrifugal impeller.

## 2. From Conventional to Miniaturized Cardiopulmonary Bypass Circuits

Two of the major innovations in CPB technology were the introduction of membrane instead of bubble oxygenators and later the use of centrifugal instead of roller pumps. In the nineties, off-pump coronary artery bypass grafting was promoted in order to avoid some of the side effects of extracorporeal circulation (ECC) and to become more competitive with the rising number of percutaneous coronary procedures. At this time only a small number of multidisciplinary teams, including surgeons, anesthesiologists and perfusionists performed “off-pump” coronary artery bypass grafting (CABG) and slightly thereafter other teams started to develop what was called a minimally invasive CPB system [1,2]. The initial concept was based on a closed-loop perfusion circuit with a non-occlusive pump. These systems have considerably developed since then and the first clinical observations were encouraging, in terms of favorable overall morbidity and reduced mortality but also because of the clinical benefits due to less microcirculatory alterations because of improved microcirculation, reduced hemodilution, better preservation of the immune reactions and less coagulation disorders [3,4,5,6].

These advances in surgical technique and in CPB technology have allowed to minimize the invasiveness of surgery and—together with improved perioperative care—made it acceptable for high-risk and aging patients, suffering often from multiple and/or severe comorbidities.

Additional technical innovations in the field of the ECC included the better biocompatibility of CPB circuits, for instance by heparin coating of the tubing systems and by reduction of the amount of foreign surfaces (shorter tubes, elimination of unnecessary components). These options helped to reduce the adverse effects generated by the plasma protein defense system and the cellular-based cytokine-mediated responses. Thereby they significantly contribute to improve clinical results.

## 3. Definition and Potential Benefits a Minimally Invasive Perfusion Systems

In the 1990’ies, discussion emerged about the potentials for reduction of the side-effects caused by ECC and the possibility to perfuse with a reduced equipment partially because off-pump surgery could not convince the entire cardiosurgical community. These discussions constituted a boost for what is called nowadays miniaturized extracorporeal systems. Nevertheless, it was rapidly shown that a miniaturized circuit is not only a reduced size system but a completely new concept of ECC. The overall goal is a constant volume perfusion with minimized blood trauma, reduced hemodilution and therefore less inflammatory response; in addition, lower transfusion requirements and accelerated early postoperative recovery are targeted.

The original miniaturized CPB systems were simplified heart-lung machines where the venous reservoir and the suckers were left off and the tubing system was significantly shortened. Heparin-coating of the tubing and the compounds is optional. A separate heat exchanger is futile since it is incorporated in the oxygenator.

Nowadays a true minimally invasive extracorporeal perfusion system consists of a closed CPB circuit that can be transformed immediately in an open circuit in case of unexpected event (massive bleeding requiring rapid re-transfusion as an example): this type of system includes as a minimal option a centrifugal pump and a membrane oxygenator. Therefore, the priming volume is significantly reduced to 300–500 mL. All other components are optional: the heat exchanger, an additional pump for cardioplegia, a venous bubble trap/venous air removing device and finally a shed blood management system. Depending on the adopted strategy, the inclusion or not of such compounds may differ from system to system [7,8].

One of the most interesting observation made when using a minimal invasive CPB circuit is the beneficial effects on the activation of the inflammatory cascade, both directly (through a blunting of the contact-phase activation) and indirectly (through a limited thrombin generation, platelet activation, and consequent lower release of pro-inflammatory cytokines). From the clinical point of view, we have observed a considerably lower rate of postoperative atrial fibrillation and need for blood transfusion while the incidence of pulmonary and renal complications was significantly decreased when a minimal invasive CPB system was used [9].

Another interesting aspect of minimally invasive CPB is its influence on the microcirculation, especially when some remnants of pulsatility (through a roller pump) are lost in favor of non-pulsatile perfusion as observed when a centrifugal pump is used in such systems. Near infrared reflectance spectroscopy (NIRS) and sublingual microscopy may be used to explore alterations in microcirculation. There is some evidence that microcirculation remains more physiological during perfusion through a minimal invasive CPB system [3,10].

In addition to all mechanical methods of reducing CPB-induced inflammation, several pharmacological approaches have been tested, including steroids, aprotinin, anticomplement strategies as well as other options to better control endothelial cell activation [11].

The main indication for miniaturized CPB system was originally coronary artery bypass grafting, where the cardiac cavities remain closed. With increasing experience, valve repair and replacement, excision of right and left atrial myxoma and simple ascending aortic replacement could be performed but required additional refinements, like introduction of a pulmonary vent that is connected to the venous return line and a smart sucker that enters in function only through an infrared light signal, which activates vacuum only once the tip enters in contact with blood in the operative field. This allows to reduce the blood-air contact even in a semi-open system [12,13].

## 4. Application in Pediatric and More Complex Adult Surgery

Based on the improved perioperative outcomes observed in adult cardiac patients, minimal invasive perfusion appears particularly attractive for smaller patients (pediatric cardiac surgery), since this particular group may significantly suffer from hemodilution.

Our initial experience with a pilot group of 38 patients in 2017–2018 using a type I circuit for closed and a type III perfusion circuits for open heart procedures was very encouraging [14]. Minimal invasive perfusion was successfully performed in all patients without technical complications nor any need for conversion. No cardiac nor neurological complication did occur in this group of patients.

Despite a clear evidence of clinical advantages in coronary surgery, the use of such systems for more complex open-heart procedures remains low [15,16,17]. Some concerns have been raised by surgeons and perfusionists regarding safety of perfusion in situations when the heart is opened and air may enter the closed system. Moreover, issues of blood and volume management have been found by some as not really practicable without having a reservoir. In the evolution of minimal invasive CPB technologies, safety aspects concerning air and volume management have been addressed by the integration of active air removal devices, and the possibility of venting and volume buffering. This has made the minimal invasive circuits suitable for valvular or even more complex aortic surgery. However, typical clinical benefits found in coronary artery bypass grafting surgery, in particular blood sparing effects, were not always reproducible. With the introduction of modular (type IV) minimally invasive systems containing a second, accessory circuit for immediate conversion to open cardiopulmonary bypass, the last obstacles have been cleared [8,18,19].

## 5. Different Types of Minimally Invasive Circuits

**Figure 1:**
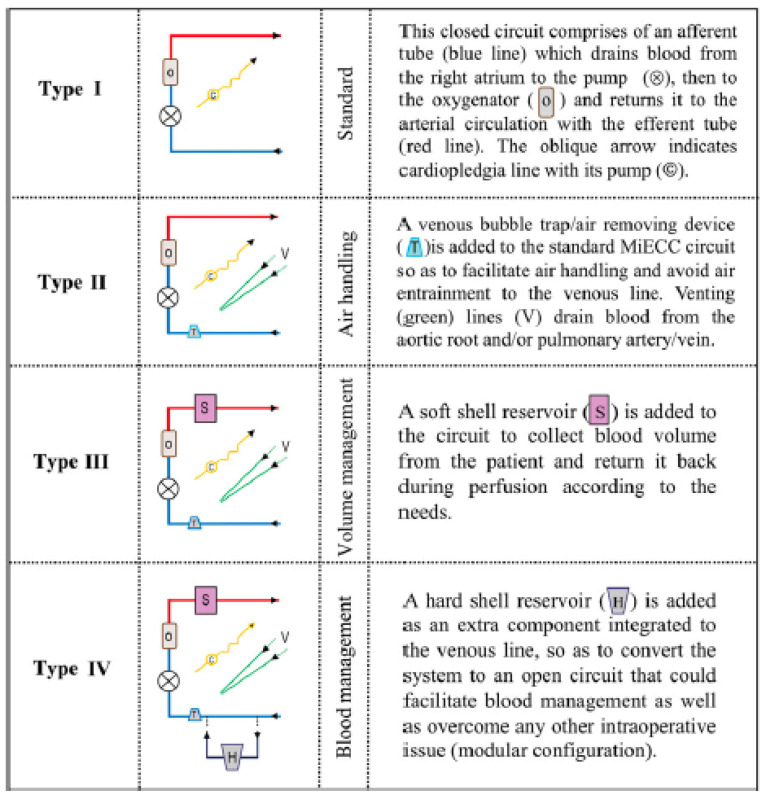
Different types of minimally invasive extracorporeal circulation systems (reproduced with permission from ref. [8]).

Over time, different types of minimally invasive cardiopulmonary circuits have been developed and are usually classified into 4 types, mainly depending from the components they include (Figure 1) [8,19].

**Figure 2:**
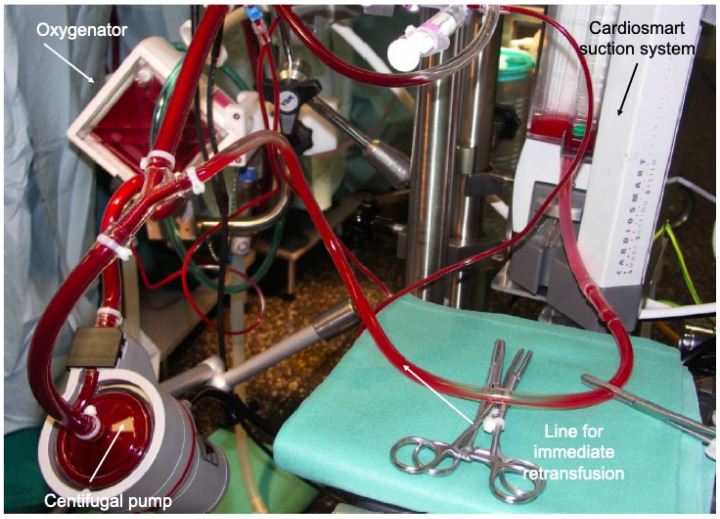
Type I mini-ECC system, including a centrifugal pump and the oxygenator. Integrated vacuum suction system (Cardiosmart©) (on the right) allowing a maintenance of a constant volume and whole blood transfusion in case of bleeding. Air removal through active handling.

Type I is the simplest one and consists of a closed circuit, which includes the oxygenator and the pump. There is no open venous reservoir. All components are coated with heparin and the tubing system is significantly reduced in length. This type of circuit is nowadays not frequently used but represents an ideal concept for patients requiring extracorporeal life support (ECLS) and for isolated coronary bypass grafting procedure. The absence of a venous bubble trap and venting lines in the original MECC system from Maquet^®^ (Rastatt, Germany) and the fact that removal or re-transfusion of shed blood is only possible with an external cardiotomy sucker is considered by some as a drawback of such a simplified system (Figure 2).

The latter constitutes the main difference between type I and type II, since type II includes a venous bubble trap or a venous air removing device (for instance the Resting Heart System^®^ from Medtronic, Minneapolis, MN, USA). With the help of ultrasound detectors, air bubbles can be recognized and automatically removed, which makes the system much safer. If necessary, a left ventricular vent can be integrated but this makes the system semi-closed. Finally shed blood can be separated and processed. In such closed circuits no shift of volume between the patient and the system is possible while the patient’s own venous capacitance serves as a volume compensation system. This means that positioning of the patient (Trendelenburg or anti-Trendelenburg) is the most efficient method to shift volume within the patient. The characteristics of type I and II circuits allow a reduction of the priming volume of between 400 and 650 mL. Anticoagulation is monitored using the activated clotting time, with a targeted value around 300–400 s. Initial heparin administration consists of 150 IE/kg.

**Figure 3:**
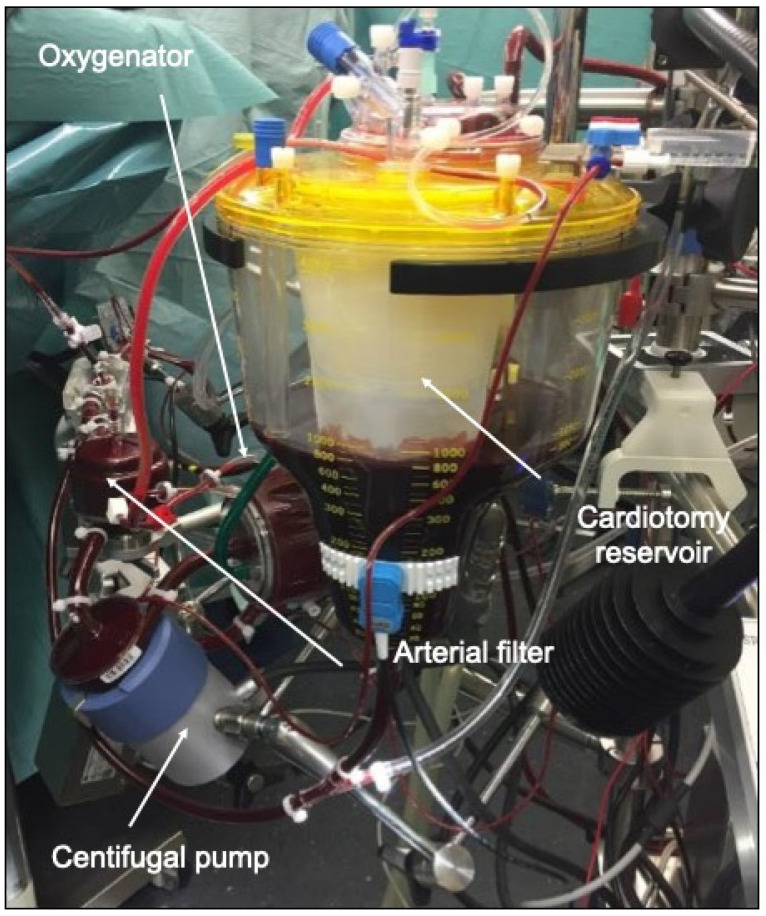
Type III mini-ECC system with a priming volume of 700 mL (not completely closed system with remaining blood/air contact).

During a true open-heart procedure like valve surgery, left ventricular venting is usually required as blood-air interaction is usual. The main additional characteristic of a type III (Figure 3) circuit is the ability to control volume shifts and variations more efficiently. This is achieved through the inclusion of a volume collection bag into the circuit and a bubble trap. In addition, one or more of the following components are usually included: a pulmonary artery vent, an aortic root vent or a left ventricular venting. This helps to operate in a blood free surgical field.

The main advantage of a pulmonary artery vent is the possibility to connect it directly with the venous return line. With this, the vent is directly dependent on the negative pressure of the centrifugal pump. However, this is also the limiting factor for vent performance. However, the speed of the centrifugal pumps has to be adjusted to the venous return, otherwise venous vascular collapse may happen and lead to unstable perfusion. In addition, the pulmonary artery vent is not a true left ventricular vent and thereby cannot properly de-air the left heart cavities during reperfusion. Immediate re-transfusion of the volume drained by pulmonary artery vent into the circuit without blood-air contact is one of the major advantages of type III. When a left ventricular vent is introduced, the drainage of the left heart cavities will be more effective but this happens with the loss of one of the major advantages of a true minimally invasive circuit, namely, the avoidance of blood-air contact. Consequently, the shed blood collected by the left ventricular vent cannot be drained directly into the circuit without the risk of risking air trapping into the system. This can be avoided by using a venous bubble trap which is introduced in the line to the soft bag reservoir for direct blood re-transfusion. Another option might be the direct but manually cross-clamped connection to a hard-shell reservoir where the volume can be intermittently re-transfused into the circuit by manual de-clamping the connection line [19].

**Figure 4:**
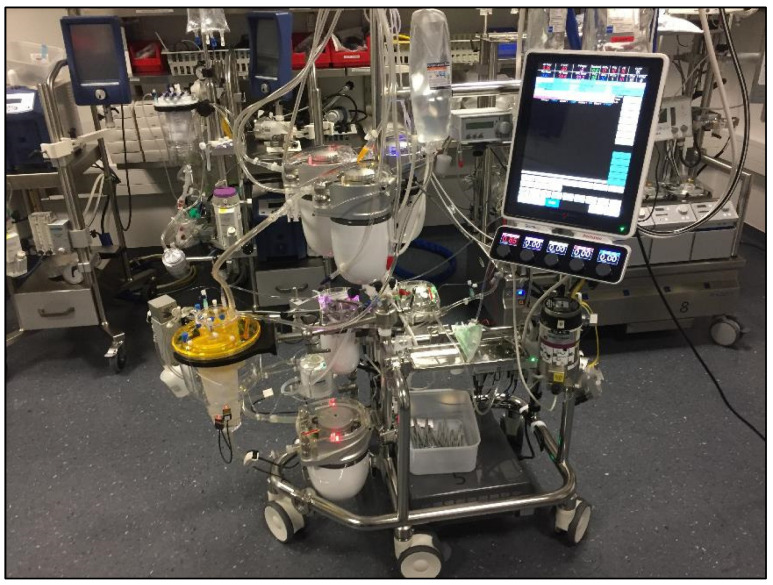
The most modern conventional ECC system with compact arrangements of all compounds.

Type IV is the most versatile type that is still considered by some as a minimized circuit; the major advantage is the possibility to convert it very instantaneously in a fully conventional CPB circuit in case of any technical trouble (for instance massive bleeding and need for immediate major re-transfusion). Critically thinking, the types III and IV diverge somewhat from the original “reductionistic” concept of what a minimally invasive cardiopulmonary circuit should be. On the other side, most recent developments in the field of ECC have led to the construction of minimized conventional circuits that also allow a significant reduction of the foreign surfaces and a minimization of the priming volume of the circuit (Figure 4).

A specific aspect of the use of minimally invasive CPB systems is the need for more intensive communication that is required between the perfusionist, the cardiac surgeon and the anesthesiologist. Since the tubing system is significantly shortened, the console of the CPB has to be placed much closer to the operating table.

## 6. Micro-Cardioplegia

One of the most interesting aspects when performing cardiac procedures using a minimal invasive perfusion system is the administration of cardioplegia. Basically, the aim of myocardial protection is to create conditions that permit a cardiac surgery procedure while myocardial integrity and function are preserved. This goal is realized by lowering the metabolic rate during cardiac arrest, creating a favorable milieu that may provide safety during arrest and finally by a controlled reperfusion to decrease structural and/or functional damage. Over the years, a multitude of strategies for myocardial protection has been used, both with crystalloid or blood cardioplegia. In addition, the technique of delivery (antegrade or retrograde) and the amount of administered volume varies considerably. Because no superiority of either delivery technique could be proven, cardioplegia administration is far from being standardized among institutions.

In patients operated with a miniaturized CPB system, there is usually no additional pump for cardioplegia. Therefore, the majority of teams have used a conventional crystalloid cardioplegia (Bretschneider, St-Thomas or Del Nido) but this usually means that 1 to 2 L of volume are infused following aortic cross-clamping. Therefore, some advantages of the mini-circuits to decreased hemodilution are lost during cardioplegia application [20,21,22].

Alternatively, cardioplegia is administered with low volume, according to the Calafiore technique: arterial blood is deviated from the main line, enriched with potassium and then returned to the venous line. To maintain the system closed, a sucker is not part of the system and the collected blood is washed through a cell saver and re-transfused by the anesthesiologist.

With increasing experience using mini-CPB circuits, we developed our own low volume cardioplegia solution, Cardioplexol™ (Bichsel, Interlaken, Switzerland. The latter is composed of potassium, magnesium and xylitol (solution A, 95 mL) and procain (solution B, 5 mL). Immediately before administration, solutions A and B are mixed (total = 100 mL) and are ready for use. Cardioplexol™ has been introduced in 2003 at the University Hospital in Bern (Switzerland), and then slightly modified [18,19]. Practical advantages including the easy and rapid administration by the surgeon him/herself, the almost immediate cardiac arrest and a protection time of 50 to 60 min have been observed. In a single centre, retrospective observational analysis of 7447 adult cardiac operations prospectively collected, 2416 were isolated coronary bypass operations performed with a miniaturized circuit. Patients were 81.3% males, 66.2 ± 9.7 years old and had a median logistic EuroSCORE of 3.2. Median cross-clamp time was 45 min and more than 75% of the patients received only one dose of Cardioplexol™ (100 mL). Following opening of the aortic crossclamp, more than 90% of the hearts spontaneously recovered a rhythmic activity [23].

Maximal value of troponin T during the first hours following myocardial reperfusion was 0.9 ± 4.5 ng/mL (median = 0.4 ng/mL). Mortality at 30 days was 0.9%. In this study Cardioplexol™ was found to be promising, because it appeared efficient and safe. In case the ischemic time is estimated to be far over 60 min, a second dose of 50–100 mL Cardioplexol™ (depending on the estimated remaining ischemic period) must be administered. The same applies if the ischemic time will be over 90 min, and so on.

During an extended period of observation, no adverse event could be directly related to the use of this cardioplegic solution. Cardioplegia with Cardioplexol™ was in our eyes particularly beneficial when minimally invasive system was used. Its application, although very simple, requires however a few words of caution: since it is often used once only, it is obvious that the solution must be administered perfectly. For this, the aortic valve should be checked for significant regurgitation since the latter would preclude the correct perfusion of the coronary arteries. In that case, selective administration should be performed directly into the coronary ostia. The presence of a ventricular hypertrophy must also be checked as a higher initial dose may then provide a better protection [24].

## 7. Coagulation Management

Point of care testing of the anticoagulation has been proposed to facilitate assessment of intraoperative patient’s coagulation status and to make subsequent adaptation more expedient. In previous works, we have advocated individualized heparin and protamine management. Low anticoagulation protocols are routinely followed when a minimally invasive circuit is used while continuous intravenous heparin infusion is preferred instead of intermittent heparin bolus administration [25]. A heparin-protamine ratio of 0.7 is used. Residual heparin levels are excluded before the patient leaves from the operating theater. Point of care coagulation management is especially helpful when diffuse nonsurgical bleeding is observed in the surgical field after heparin reversal because it allows expedient correction in the operating theater.

In cases with severely impaired coagulation because of hypothermia or any other adverse condition (emergency, preoperative anticoagulation or anti-aggregation), thrombo-elastometry allows for precise assessment of a patient’s coagulation status even in the presence of full heparinization before weaning from CPB. This will significantly reduce the so-called bleed-to-treat time. In case of fibrinogen deficiency, fibrinogen concentrate or an appropriate amount of fresh-frozen plasma (FFP) or cryoprecipitate is administered. Prothrombin complex concentrates are administered in case of a significantly prolonged clotting time without evidence of residual heparin or fibrinogen deficiency, or as a second line in the case of a prolonged clotting time after fibrinogen/FFP supplementation. If nonsurgical bleeding after heparin reversal is observed despite adequate fibrinogen levels, aggregometry should be evaluated. In case of low values ADP and TRAP tests, platelet transfusion should be considered. Minimally invasive perfusion systems should make possible to adopt a more restrictive policy of foreign blood transfusion anyway [26].

## 8. Clinical Experience

Several studies have described the advantages of minimally invasive extracorporeal perfusion [15,16,17,26,27]. One of the rare prospective randomized multicenter trial hypothesized that minimally invasive extracorporeal circulation would reduce the risk of serious adverse events after cardiac surgery operations [28].

The study was conducted in patients undergoing elective or urgent isolated coronary artery bypass grafting, isolated aortic valve replacement surgery, or combined coronary and aortic valve surgery [29]. The primary outcome was a composite of 12 post-operative significant adverse events up to 30 days after surgery, the risk of which minimized circuits was hypothesized to reduce. Unfortunately, the trial was terminated early due to the COVID-19 pandemic [24]. 1071 participants with median Euroscore II 1.24 were randomized. 50 of 517 (9.7%) randomized to minimized circuits and 69/522 (13.2%) randomized to conventional CPB experienced the primary outcome (risk ratio = 0.732, *p* = 0.025). The risk of any significant adverse event not contributing to the primary outcome was similarly reduced (risk ratio = 0.791, *p* = 0.250). The potential benefits of minimally invasive cardiopulmonary bypass remained uncertain because the trial did not achieve the target sample size (29).

Nevertheless, the most recent developments in CPB technology go in direction of improving and simplifying the conventional circuits as well; in that term, the differences between a type III or IV minimally invasive circuit and the most modern conventional CPB (Figure 4) circuit are continuously shrinking.

Although there are several differences between miniaturized and conventional CPB circuits, potential advantages and disadvantages are difficult to be detected; in addition, the large heterogenicity of the systems used in clinical practice makes comparison difficult.

The foundation of the multidisciplinary Minimal Invasive Extracorporeal Technologies International Society (MiECTiS) in 2014, constituted a new era in perfusion with the aim to build up a group of experts interested in refining the technology and implementing it into clinical practice. Another important aspect was to promote a common language between all specialties involved, such as cardiac surgeons, anesthesiologists and perfusionists. The position paper published in 2016 and updated in 2022 was a collaborative effort to set standards for definition of mini-CPB systems and to provide evidence-based recommendations for the major European scientific societies in the field of cardiovascular surgery (8,19). This group of experts also designed the randomized controlled trial to evaluate minimal invasive versus conventional ECC [28]. The results of this study will be published soon (29).

## Data Availability

Not applicable.
